# Secondary Guilt Syndrome May Have Led Nazi-persecuted Jewish Writers to Suicide

**DOI:** 10.5041/RMMJ.10225

**Published:** 2015-10-26

**Authors:** George M. Weisz

**Affiliations:** Senior Lecturer, School of Humanities (Program in History of Medicine), University of New South Wales, Sydney, Australia, and University of New England, Armidale, New South Wales, Australia

**Keywords:** Guilt, persecution, suicide, survival, writers

## Abstract

Feelings of guilt have tormented Holocaust survivors, ranging from immediately after the liberation to later in life, for shorter or longer periods, and persisting for some throughout their entire post-war lives. Descriptions of the guilt experienced by survivors of the Nazi camps occupy an impressive amount of literature: “Why me?” was the question, when a younger and more able family member perished; “Why me?” when more productive members of the community perished; “Why me?” when a million and a half children were deprived of their lives. Many found the answer by retelling their stories, witnesses of what happened. This type of guilt is much different from the recently described phenomenon of survivor syndrome, namely the secondary guilt felt by Nazi-persecuted Jewish writers. Despite successes in all aspects of their life, these writers developed a self-incriminating guilt due to their perceived inadequacy of communicating, particularly in light of the resurging anti-Semitism worldwide. This paper deals with the survival and suicides of Nazi-persecuted Jewish writers and offers a possible explanation for their late self-destructive acts.

## INTRODUCTION

The problem of guilt experienced by survivors of the World War II (WWII) concentration camps is well-documented.^1^,^2^ In answer to the question of “why,” many survivors found meaning in statements such as “I survived so that I could bear witness.”^3^ This response is simplistic, however, for Jewish writers who survived the camps.

To establish a baseline, the case of Jewish writers in Central European lands is illustrative. Emancipated by the Enlightenment, Jews in Central Europe flooded the universities in the late eighteenth and early nineteenth centuries, first as students and then as teachers. Once Jewish writers were permitted to contribute to the literary world, their number grew and proportionally exceeded that of their brethren in the general population. Many writers acquired a perfect command of, and developed a deep love of the vernacular language, none more so than the German one.

The disseminated theories on post-Auschwitz writings have varied: some have stated that too much was written, others that not enough was written. Perhaps the most intellectual of writers who survived the Holocaust, Jean Amery (whose perspective was supported later by the intellectual Primo Levi), defined an intellectual’s qualities: “one was not merely educated, but had an aesthetic consciousness, with humanistic and philosophical views cultivated in many spheres.”^4^ Such intellectuality proved to be a disadvantage in the Nazi camps.^5^ Looking back, Amery believed that post-Holocaust writing needed to theorize more on that “universe” which he had survived. This paper looks more deeply at that “universe” and its impact on Jewish writers who survived the Holocaust.

## MATERIALS AND METHODS

The encyclopedia of post-Enlightenment, the Metzler Lexicon of German-Jewish writers, records a total of 268 Jewish writers (of whom 43 were women).^5^ Interestingly, this Lexicon enumerates only 13 Jewish writers in the eighteenth century (including 1 woman), 45 writers in the nineteenth century (with 8 women), and no fewer than 210 in the 20th century (36 of whom were women). The “writers” classification includes novelists, journalists, playwrights, art critics, art historians, essayists, and philosophers. This impressive progress slowed down in 1933, due to the restrictions initiated by the Third Reich, and ceased altogether in 1935 with the enactment of the Nuremberg Laws.

This and other relevant literature was reviewed that related specifically to the lives of Jewish writers, pre- and post-Holocaust, as well as a few writers of non-German or non-Jewish origin who had endured the same pattern of suffering. Biographical information found in the literature was searched to identify those writers who had committed suicide.

## RESULTS

The pre-Third Reich records indicated that very few Jewish writers in Germany had committed suicide. Noteworthily, all of the writers who had committed suicide during or after the Nazi regime were victims of persecution or incarceration.

### Suicide in Pre-war Germany

The eminent historian Konrad Kwiet studied the overall Jewish experience in pre-Nazi Germany. He concluded: “Organized persecution had a profound effect on Jewish morale. ... a suicide curve would document the close correlation between persecution and suicide.”^6^ His comprehensive essay shows that during the nineteenth and the first half of the twentieth century the incidence of suicide by German Jews was much lower than for the other two dominant religions (Protestant and Catholic Christianity). There were, however, occasional upsurges of suicide before WWII that paralleled periods of persecution. In these periods “Jews were driven to despair,” and their suicide rate would rise to the level of “a mass phenomenon.”^6^

Two patterns were observed in the suicides of the pre-war/war period. The first pattern related to writers who remained on German soil and were persecuted and restricted in their careers, with some being temporarily interned. These writers, unwilling or unable to leave the country, found escape in self-destruction. Examples of such were John Höxter and Ludwig Fulda, both in Berlin, and Egon Friedell in Vienna, all of whom committed suicide just before WWII.

The second pattern related to writers who had left Germany and suffered from what Roden defines as the “crisis of exile.”^7^ In 1933, and even more so after the Nuremberg Laws were enacted, Jewish writers in Germany were barred from their literary pursuits. Essays were no longer published, art critiques remained as drafts, plays went unproduced, and books were blacklisted and later thrown out of libraries and burnt on the streets. These experiences were soon augmented by fear for their lives.

Several of these writers managed to escape to Palestine, such as A. Koestler, or to free countries in Europe, the Americas, Asia, or Australasia (e.g. Walter Benjamin escaped to Spain, Stefan Zweig to Brazil, Klara Blum to China, and Karl Wolfkehl to New Zealand). For some, suffering took the form of a “crisis of exile” (*Exilkreisen*), a term that was coined by Klaus Mann and led to “*Kreisenliteratur*.”^3^,^7^ Yet another author from this second group raised the question, “How much home does a person need”?^5^

The loss of homeland with its customs, food, clothing, music, entertainment, stories of childhood, and literature made the exile not just merely physical, but cultural. “One ages badly in exile, because a human being needs a home.”^6^ These writers felt so isolated that they could not adapt to a new language.^7^

Table 1 provides a selected list of pre-war/war period writers who had suffered from “crisis of exile” and who committed suicide.

**Table 1:** Selected List of Jewish Writers Who Committed Suicide in the Pre-war/War Period.

Name	Type of Writer	Year of Suicide
*Kurt Tucholsky	Satirist, novelist, playwright	1935
*Egon Friedell	Theatre critic, journalist	1938
*John Höxter	Theatre critic, playwright	1938
*Ludwig Fulda	Playwright, author, journalist	1939
Ernst Toller	Historian, playwright, novelist	1939
Walter Benjamin	Philosopher, author, essayist	1940
Walter Hasenclever	Novelist, historian	1940
Stefan Zweig	Novelist, essayist, playwright, librettist	1942
Alfred Wolfenstein	Historian, anthologist, playwright (last published in 1936)	1945
Paul Federn	Psychoanalyst, editor, author, critic (last published in 1941)	1950

* Although these writers died in Vienna and Berlin, “crisis of exile” was expressed by their literal fear of exile, which led to their suicides.

### Suicide during World War II (1939–45)

Intellectuals in the Nazi camps were unique, as they suffered more than average people, both during their early detention and their later incarceration in the “*univers concentrationnaire”*^4^,^7^ (i.e. concentration camp universe). The harshness of unaccustomed manual work, the hunger, the cold, the crudeness of non-existent hygiene, and the personal humiliation were made more difficult and painful as they were singled out for particular persecution by their tormentors. Paradoxically, the desire to commit suicide seemed to diminish during camp life: “Suicide was not a typical response to the concentration camp inmate.”^7^ Instead, the daily fight for survival was paramount. The impact on their physical and mental health, however, was intense; some perished in the camps, others did indeed commit suicide.^8^

### The Post-war Period

How was it possible for these sensitive intellectuals to survive after their horrendous experiences? They did indeed have difficulties in adjusting to freedom. Birkenau camp prisoner no. 31661 (Charlotte Delbo) provides this insight into the survival syndrome: “one doesn’t die from grief—you go on living” and “I took leave of my skin—it had a bad smell, worn from all the blows it had received.”^9^ This non-Jewish French writer, arrested for anti-fascist activities, described how she “suffered survival” in the years after liberation. “I lived next to Auschwitz …, but had to return to gestures belonging to another earlier life, the using of a toothbrush, of toilet paper, of a handkerchief, of a knife and fork, and … later on, smiling.” Perhaps as atonement, she wrote the poem: “Prayer to the Living to Forgive Them for Being Alive.”^9^

The persistence of the torment and the incurability of the concentration camp syndrome were perceptively described by Kaminer: “Even if the survivors founded a new family, even if they experienced professional success, even if they have a peaceful environment, deep inside them lurks anxiety and feeling of being in constant threat … a feeling of living on razor’s edge.”^10^

Surprisingly, for writers, the release from the camps into the general community proved stressful.^1^,^11^ Suicide became more frequent, born out of a “guilt relegated to the background only to re-emerge after the liberation.”^3^ Despite their experiences, some of the surviving writers became productive. Although living in distant lands, often changing their names, most wrote in German, though a few wrote in Italian (e.g. Primo Levi), Ukrainian (e.g. Piotr Rawicz), Polish (e.g. Jerzy Kosinski), or partly in English (e.g. Arthur Koestler). Eventually, they achieved international recognition for their talents and were flooded with accolades from their peers. Amongst those who survived the camps, demonstrated some common manifestations of persecution, and committed suicide later on were: Jean Amery, Bruno Bettelheim, Paul Celan, Jerzy Kosinsky, Primo Levi, Arthur Koestler, Piotr Rawicz, Peter Szondi, and Joseph Wulf.

### Primary Guilt Syndrome

The general frame of survivor syndrome has been detailed by Niederland.^2^ In trying to explain the post-war behavior and eventual fate of writers haunted by the Nazis or their collaborators, one can use the ideas advanced by prisoner no. 174517 (Primo Levi), born out of his ordeal in Auschwitz. In 1947, Levi was one of the earliest writers to mention the “guilt of the survivors,” trying to throw some light on the differences between the “drowned” and the “saved.”^1^ He described the remorse of the survivor, for being the fittest, his survival perhaps being to the detriment of a weaker or an ignored inmate.

A very detailed study was also given by Ryn on the “Evolution of Mental Disturbances in the Concentration Camp Syndrome (KZ-Syndrom)”.^12^ In his view it was an “encephalopathic syndrome,” a multiform psychopathological disease, indicating that the syndrome is chronic and progressive.

### Secondary Guilt Syndrome

Secondary guilt syndrome of Jewish writers is presented as a psychological decompensation, following a period of success and life rebuilt. The post-war condition emerged in three stages: latency, activity, and finally secondary guilt syndrome. The initial period of literary silence (the latent period) was not the result of amnesia. For different writers, different events broke the silence: “The experience was not forgotten or repressed, it needed appropriate time when ‘suddenly everything demanded telling’.”^4^

Once this latency period had ended, commonly a period of intense literary creativity followed, which focused on Holocaust-related topics. The duty to bear witness to the atrocities was a compulsive driving force for many survivors, and more so for the intellectuals, including gentile prisoners like Terrence Des Pres^13^ and Charlotte Delbo.^9^

The last stage of the writers’ suffering, secondary guilt syndrome, began to manifest via a disappointment that led to a pre-suicidal period. Once they felt that they had fulfilled their duties and had disseminated their testimonies, they suddenly sensed that their audience was tiring of the subject. “The survivors wanted to transmit a message … but you discarded their testimony.”^14^ Their perception of an indifferent world led to catastrophic thinking, to dysfunctional periods in their lives, and to the uncontrollable “psychache” studied by Janet McCord.^15^ Their post-Auschwitz period of life was an “existence only.” Being incapable of finding joy anymore, they felt defeated by the world’s attitude. Once they reached “the end of [their] road” they eventually “drowned” themselves.^16^

The Jewish writers discussed herein survived the Holocaust, passed through these three stages, and eventually committed suicide. Three of them are described in more detail below.

#### Jean Amery

The story of the Austrian writer Jean Amery was typical. Born as Hans Mayer in Vienna in 1912 to a Jewish father (killed fighting for the Austro-Hungarian Empire during World War I) and a Catholic mother, he studied philosophy. He fled his native land in 1938 after the annexation of Austria. Changing his name to Jean Amery, he settled in Belgium. Amery was arrested for anti-fascist activities and severely tortured. Once it was learned that he was Jewish, he was sent to Auschwitz and later to Buchenwald. In 1945, Amery was one of the walking skeletons found by the British in the liberated Bergen-Belsen. After regaining his “freedom,” Amery lived in Belgium. He was silent for some 20 years, until “I could again meet Germans impartially.”^17^

His autobiographical stories (essays, critiques, and books) were not published until 1966, when he could finally write about “things that were weighing on my soul.”^17^ His writings were retrospective analyses of his experiences influenced by contemporary attitudes. Amery frequently talked and wrote about death and suicide. He wrote that his life was “an existence only.”^18^ He must have been aware of his mental state: “I was told of my mental damage apart from the physical, it was called ‘concentration camp syndrome,’ a ‘delayed psychic effect’.” He stated: “I neither can nor want to get rid of resentment.” Amery’s awakening came “when the old anti-Semitic attitudes re-emerged on the world scene” in parallel with Germany’s grandiose resurrection; it was a “loss of trust in the world.”^18^

His feelings of guilt were based on the perception of his inability properly to communicate his experiences. For Amery, aging was made particularly painful “by scars left by the Auschwitz experience.”^18^ Amery analyzed the resurgence of anti-Semitism and rejected the idea of “forgiving or forgetting.” His last autobiographical books were “Lean Journeyman Years” (1977) and his posthumous “Carry On—But How?” in 1982.

**Figure 1:**
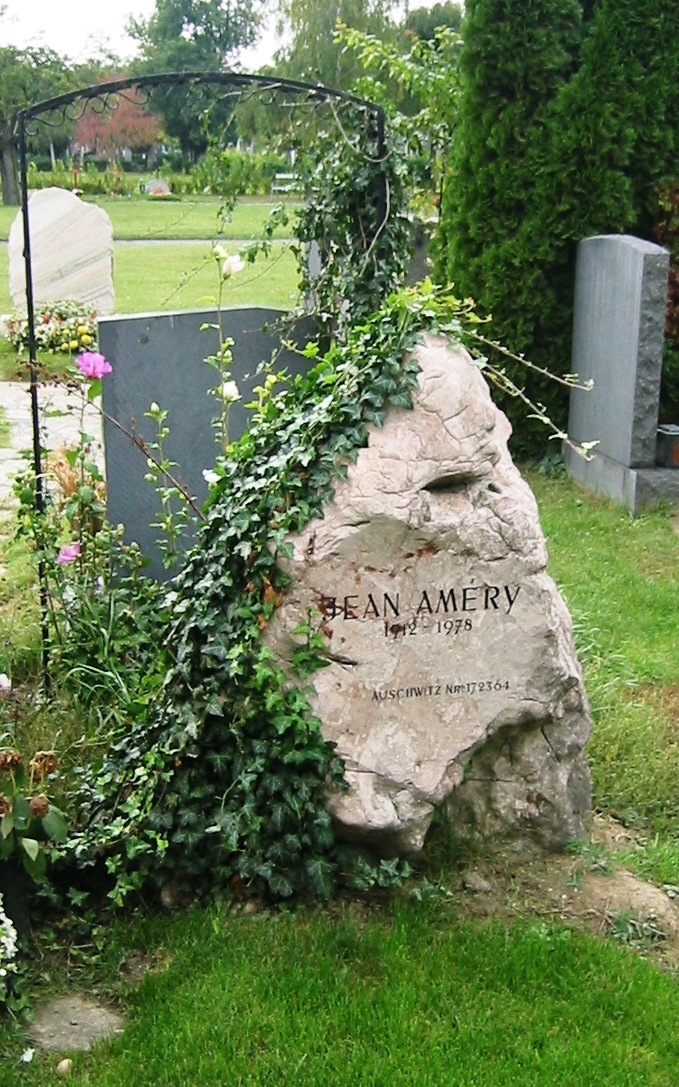
Grave of Jean Améry at the Zentralfriedhof Vienna Photography by Invisigoth67 under a CC BY-SA 2.5 license (https://en.wikipedia.org/wiki/Jean_Am%C3%A9ry#/media/File:Ehrengrab_Jean_Amery.jpg).

Amery committed suicide in 1978. Was his suicide a sign of unrecognized and untreated depression, which eventually with age and deteriorating health led him to take “the last path to freedom”?^5^ Appropriately, his grave in Vienna is marked by a simple, unpolished rock, inscribed with his name, year of birth and death, and “Auschwitz Nr 172364” (Figure 1).

#### Primo Levi

Primo Levi’s story is particularly tragic. He was born in Torino, Italy, in 1919 into a large, assimilated, and educated family that gave the world artists, scientists, and a Nobel Laureate. Primo studied organic chemistry, barely managing to finish his studies before the Racial Laws were enacted in Italy. Aged 20, he joined the anti-fascist movement and was captured and subsequently interned in the camp for political prisoners. Betrayed by a fellow partisan as being Jewish, Levi was sent to Auschwitz as prisoner no. 174517.^19^ Physically frail but intellectually alert, Levi managed to survive for 10 months in Auschwitz. After liberation, reunited with his family, he soon started work in an old chemical factory, married, and had two children. His career as a writer was slow to emerge, and success came late. His fame and the love of family and friends did not suffice, however; he could not forget what had happened. Indeed his teachings and lectures were always on Holocaust topics. In 1982 Levi stated: “to deny Auschwitz is to be ready to rebuild it.”^20^

His feelings of guilt were based on the perception that he had insufficiently communicated his message, thereby allowing the world to become indifferent to the past. His emotional disturbance became steadily more pronounced, culminating in his plunge to death on April 11, 1987. Levi lived “without peace,” passing through all three phases of post-war suffering. The author Elie Wiesel asked, “Why Death, Primo?” and then answered his own question: “He killed himself because he could not go on.”^21^

Levi was known to have suffered from repeated bouts of depression. He was treated for at least six years by a psychiatrist, a psychoanalyst, and a neurologist. He was also treated with a monoamine oxidase inhibitor, tramylcypromine (Parmodalin) with trazodone (Trittico or Desyrel), sleeping tablets (Neulactil), and anti-dementia medication.^22^,^23^

**Figure 2:**
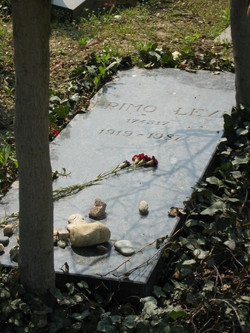
Grave of Primo Levy Photo from http://www.findagrave.com; reproduced with permission of the photographer, Laura James.

The officially declared suicide was questioned by some friends. However, none contested his severe depression, perhaps aggravated by several somatic disorders. Levi’s words were: “... the most serious disease I ever had: the tattoo on my forearm.”^23^ Appropriately the simple stone on his resting place is marked with his prisoner number and years of birth and death (Figure 2).

#### Joseph Wulf

**Figure 3:**
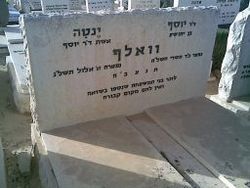
Grave of Joseph Wulf

Joseph Wulf was born in Chemnitz, Germany, in 1922 and received a rabbinical education. He experienced ghetto life, and at the age of 22 he became prisoner no. 114866 in Auschwitz. After the war, he wandered through Poland, Paris, and Berlin. His driving force was “to do everything to prevent the world from forgetting the millions of murdered Jews.” He was silent for 14 years, after which he wrote many books—all of which were only about the Holocaust. At the end of his life, he lamented in a letter to his son: “I have published 18 books about the Third Reich and they had no effect ... mass murderers walk around free, live in little houses, and grow flowers.”^24^ He fought for the creation of the Wannsee Memorial in the Berlin villa where the Final Solution was decided upon.^25^ In October 1974, Wulf jumped to his death. Although he died in Germany, his final resting place is in Israel, where he is buried beside his wife; appropriately, the stone is marked in Hebrew with his name (Figure 3). Clearly, Wulf’s feelings of guilt were based on the perception that he had failed to have a positive effect in remembering the Holocaust, or in bringing justice to the perpetrators.

## DISCUSSION: SUICIDE, A SOCIAL PHENOMENON

As mentioned above, the Jewish intellectuals suffered more in the camps. They were recognized, detested, and singled out by the guards, and punished and beaten more by both envious kapos and the SS. The intellectuals were persons whose main activity was the production or evaluation of ideas;^26^ for some, the only way out of their mental suffering was self-destruction.

The phenomenon of the interdependence between suicide and the social environment was already established in 1897 by the sociological studies of Emile Durkheim.^27^,^28^ The present paper suggests applying this sociological theory to the circumstances of the Nazi period. There are no precise statistical documents on the suicide rate during the Third Reich period. However, estimates indicate that suicides were in the thousands, peaking in the period of the Jewish boycotts such as the 1938 annexation of Austria and the November 1938 nationwide pogrom.^29^

Incarceration caused prolonged psychic stress and severe physiological damage and even metabolic deprivation with its secondary organic brain syndrome. It also had a chronic effect on a nervous system already damaged by famine and injury. It has already been medically established that these factors predispose humans to emotional decompensation.^30^

The example of persecuted Jewish writers serves as an extension of the above-mentioned social thesis of suicide. The biographical data on Jewish writers who committed suicide reveal that all were persecuted or incarcerated, some were exposed to famine, to severe loss of weight, and yet others to the cold and to physical abuse. No study has yet shown if there was a higher incidence of suicide among Jewish writers compared to non-Jewish writers, or compared to survivors in general. Likewise, no data have correlated the type of suicide with the type of personality, or explained the gender dominance. No studies have been made of their childhood or adolescent life to discover a potentially predisposing personality. However, just as these intellectuals suffered in the camps, they faced difficulties in adjusting to the free world.

The initial guilt they experienced was for surviving—a tormenting remorse that remained for years. That feeling of guilt was further amplified by the delayed, self-incriminating guilt of failure as communicators. The parallel rise of anti-Jewish hatred led to their “ultimate refuge,” a delayed form of homicide committed by their persecutors.

This extension of Durkheim’s theory to the Nazi period has been supported by the non-Jewish writer Tadeusz Borowski (Auschwitz no. 119198), who underwent the same pattern of suffering and suicide. He described his experiences in his book “This Way for the Gas, Ladies and Gentlemen,” published just three years before his suicide.^31^

What was different in the case of those writers who did not “drown?” Indeed, there were persecuted or incarcerated writers who did survive, who became productive, and who made no known attempts at suicide. One can only speculate about how survivors like Viktor Frankl, Samuel Pisar, Simon Wiesenthal, and Elie Wiesel were “saved” from that tragic end. There is no universal explanation.

An important observation has been raised by Amery: namely that ideology and faith were an “inestimable help ... under conditions that defy the imagination: Catholics performed Mass, Orthodox Jews fasted on the Day of Atonement, the Marxists persevered that the Soviet Union will and must win.”^5^ A similar view on this phenomenon was offered by Porter^32^ and Rudolph,^33^ who described the more strongly motivated inmates as those with either a political conviction or religious beliefs—in Porter’s words “belief in God was a life support … being religious is healthy.”^32^

It was the view of Elie Wiesel (a survivor of Auschwitz and Buchenwald, and a post-Holocaust Nobel Peace Prize Laureate) that it was easier to survive in camps for those with either a political ideology (communists, Zionists) or religious belief. In the Lexicon studied for this paper, there is no evidence of religious attachment among any of the listed suicidal writers.

One might ask: was religion the life jacket that prevented Elie Wiesel from “drowning?” That might just be the case, as when reading the suggested concept on Secondary Guilt, Elie Wiesel accepted it as being “painful and insightful” (personal letter to the author, dated September 16, 2002).

This paper has sought to extend Kwiet’s study of suicide in Germany beyond the WWII period. Applying Durkheim’s social theory of suicide, it has been suggested that Nazi persecution was the direct cause of the guilt experienced by persecuted intellectuals. Following the initial and primary guilt of the survivors, this feeling was later amplified by a perceived failure in their duty to communicate. Certainly Bettelheim and Primo Levi, in choosing the anniversary of their liberation as the day of suicide, expressed a firm connection between persecution and suicide.^34^

Durkheim stated that, “… the Jew is an intellectual and a man of culture … has all the intelligence of modern man without sharing his despair.”^28^, ^p.167^ This might be true; however, it does not seem to apply to Nazi-persecuted Jewish intellectuals. Durkheim’s work did not anticipate what actually happened: that the successful fight for survival in the camps would lead to a continuous fight for survival post-war. Would the famous sociologist have expected the continuous survival of these Jewish intellectuals—all of whom developed successful careers and were offered accolades, won prizes, settled with families, and had no financial problems—without giving in to “despair?” This question will have to remain unanswered.

It is suggested that although Primo Levi threw himself to his death, he was “pushed” by this delayed secondary guilt syndrome. The chief rabbi of Torino, Emanuele Artom, clearly supported this concept by permitting Primo Levi to be buried in the Jewish cemetery, contrary to tradition. In the Rabbi’s view, Levi’s death was not truly suicidal self-destruction, but rather a delayed Nazi homicide.^23^

The concept that the suicide of Jewish intellectuals may be a delayed homicide by the Nazis rather than an act of self-harm is controversial. Many psychologists and historians would dispute it. However, it seems to the author as plausible from a medical perspective, and it is certainly different from post-traumatic stress syndrome. However, it would be simplistic to accept these suicides within a mono-causal explanation. The perceived inability to express their fear of the resurfacing hatred and their perceived impression of failure to communicate the uncommunicable (in the case of e.g. Bettelheim) persisted. Their guilt for failing convincingly to warn the world of the early signs indicating a re-emergence of old, temporarily suppressed ideas may well explain their suicides—their last way of exiting from a persisting “psychache.”

## CONCLUSION

The writers discussed herein suffered from self-incrimination and developed a two-fold sense of guilt: the first for their inability to communicate the horrors of what had been experienced in the camps, and the second for their inability to prevent the atrocities they had witnessed and their reoccurrence. Theirs was a prophetic guilt that feared the resurgence of a similar hatred. Indeed, this dormant anti-Semitism has exploded in our decade, coincidentally soon after the demise of these writers. They were persecuted because they were Jews, and they died because they were Jews. Hence, their perceived sense of guilt has been considered as quite real by the Jewish community, and it was the effect of their suffering as Jews that led to the decision that they deserved to be buried with their brethren.

It seems symbolic that Levi and Bettelheim, as mentioned above, ended their lives on the anniversary of their “liberation.” Another sensitive intellectual, Doctor of Medicine, Arthur Kassel, likewise committed suicide on an anniversary, in his case on the eight-year anniversary of his being forced to leave his family behind in Germany. Unable to save them, he killed himself upon learning of their final end in Auschwitz. He too was deemed a victim in 1947 and appropriately buried in the Jewish Cemetery in Sydney.^35^

## Abbreviations

WWII: World War II.

## References

[1] Levi P (1996). Survival in Auschwitz.

[2] Niederland WG (1968). Clinical observations on the “survivor syndrome”. Int J Psychoanal.

[3] Levi P (1989). The Drowned and the Saved.

[4] Amery J (1980). At the Mind’s Limits.

[5] Kilcher AB (2000). Metzler Lexikon der deutsch-judischen Literatur.

[6] Kwiet K (1984). The Ultimate Refuge.

[7] Roden R (1982). Suicide and holocaust survivors. Isr J Psychiatry Relat Sci.

[8] Ryn Z (1986). Suicides in the Nazi concentration camps. Suicide Life Threat Behav.

[9] Delbo C, Langer L (1995). “Voices” from Days and Memory. Arts from the Ashes A Holocaust Anthology.

[10] Kaminer IJ (1996). On Razor’s Edge. Fritz Bauer Institut Auschwitz Geschichte, Rezeption und Wirkung.

[11] Lester D (1986). Suicide: the concentration camp and the survivors. Isr J Psychiatry Relat Sci.

[12] Ryn Z (1990). The evolution of mental disturbances in the concentration camp syndrome (KZ-syndrom). Genet Soc Gen Psychol Monogr.

[13] Des Pres T (1976). The Survivor: An Anatomy of Life in the Death Camps.

[14] Wiesel E (1978). A Jew Today.

[15] McCord J (1994). The psychache. J Psychol Judaism.

[16] Raggam M (1973). Walter Hasenclever: Leben und Werk.

[17] Amery J (1989). Radical Humanism.

[18] Amery J (1994). On Aging: Revolt and Resignation.

[19] Anissimov M (2001). Primo Levi: Tragedy of an Optimist.

[20] Levi P, Belpoliti M, Gordon R (2001). The Voice of Memory: Interviews 1961–1987.

[21] Wiesel E (2000). And the Sea is Never Full: Memoirs, 1969.

[22] Thomson I (2002). Primo Levi.

[23] Angier C (2002). The Double Bond Primo Levi: A Biography.

[24] Lehrer S (2000). Joseph Wulf. The Creation of the Wannsee Memorial. From the Wannsee House and the Holocaust.

[25] Lehrer S (2000). Wannsee House and the Holocaust.

[26] Calhoun C (2002). Dictionary of the Social Sciences.

[27] Bettelheim B (1960). The Informed Heart: Autonomy in a Mass Age.

[28] Durkheim E (1950). Suicide.

[29] Kwiet K, Pehle WH (1938). To Leave or Not to Leave: The German Jews at the Crossroads. From Kristallnacht to Genocide.

[30] Eitinger L (1979). Concentration Camps Survivors in Norway and Israel.

[31] Borowski T (1967). This Way for the Gas, Ladies and Gentlemen.

[32] Porter JN, Cargas HJ (1999). Holocaust Suicides. Problems Unique to the Holocaust.

[33] Rudolph A (1990). At an Uncertain Hour: Primo Levi’s War against Oblivion.

[34] Fischer DJ (2008). Bettelheim: Living and Dying.

[35] Weisz GM (2015). Remembering Jewish physicians. Isr Med Assoc J.

